# Identification of Behavior Change Techniques From Successful Web-Based Interventions Targeting Alcohol Consumption, Binge Eating, and Gambling: Systematic Review

**DOI:** 10.2196/22694

**Published:** 2021-02-09

**Authors:** Gabrielle Humphreys, Rebecca Evans, Harriet Makin, Richard Cooke, Andrew Jones

**Affiliations:** 1 School of Psychology University of Liverpool Liverpool United Kingdom

**Keywords:** systematic review, web-based intervention, behavior and behavior mechanism, behavior change technique, alcohol consumption, binge eating, gambling

## Abstract

**Background:**

Web-based interventions are thought to overcome barriers to treatment, such as accessibility and geographical location, which can undermine the effectiveness of traditional face-to-face interventions. Owing to these features, researchers are increasingly testing the efficacy of web-based interventions as ways to reduce alcohol misuse, binge eating, and gambling. However, many web-based interventions have poorly defined mechanisms of action; therefore, it is often uncertain how they propose to bring about behavior change.

**Objective:**

This systematic review aims to identify effective behavior change techniques (BCTs) present in web-based interventions aimed at reducing alcohol consumption, binge eating, or gambling.

**Methods:**

This systematic review covered research conducted in the last 20 years. Inclusion criteria for interventions were web-based administration; targeting alcohol use, binge eating, and/or gambling; and reporting on baseline and postintervention measures of behavior. The PRISMA (Preferred Reporting Items for Systematic Reviews and Meta-analyses) guidelines were followed. We coded intervention effectiveness, study quality, and BCTs present in the interventions.

**Results:**

Following removal of 4152 ineligible articles, 45 were included in the review: 32 (71%) targeted alcohol misuse, 6 (13%) targeted binge eating, and 7 (16%) targeted gambling. In total, 5 frequency counts were performed to identify the most commonly used BCTs: all studies, effective interventions, high-quality studies at 2 thresholds, and both high quality and effective studies. The results obtained from this were integrated to identify 7 BCTs. These 7 BCTs were problem solving, feedback on behavior, self-monitoring of behavior, self-monitoring of outcomes, instruction on how to perform a behavior, information about social and health consequences, and social comparison. A total of 4 BCTs were found in all frequency counts: feedback on behavior, self-monitoring of behavior, instruction on how to perform a behavior, and social comparison. Self-monitoring of outcomes of behavior was found in 3 of the 5 frequency counts, problem solving was found in 2 frequency counts, and information about social and health consequences was found in 1 frequency count.

**Conclusions:**

This systematic review identified 7 of the most frequently used BCTs used in web-based interventions focused on alcohol misuse, binge eating, and gambling. These results can inform the development of evidence-based eHealth interventions that have the potential to lead to effective, positive behavior changes in all 3 areas.

## Introduction

### Background

Alcohol misuse, binge eating, and gambling are increasing in prevalence in Western countries [1-3]. Importantly, these behaviors often occur independently of a formal diagnosis of a relevant behavioral or substance use disorder, or an eating disorder in subclinical populations. This may be due to the general acceptability of these behaviors, compared with other addictive behaviors [4]. These attitudes may be explained by the legality of these behaviors and substances and their widespread accessibility. In turn, this may normalize these behaviors, something that may be problematic in individuals displaying concerning behavior in these areas. As such, many otherwise *healthy* individuals will indulge in one or perhaps all of these behaviors over the course of their lifetime. Furthermore, these behaviors can cause considerable mental and physical harm to an individual (eg, liver disease, pancreatic disease, impaired cognition [5]) and may exacerbate mental or physical health disorders. Highlighted in the UK National Health Services’ Long Term Plan (2019) [6], the effect of these behaviors will also disperse into wider society, placing further strain on health care systems [7].

Despite the introduction of various regulations, alcohol remains a highly accessible and an attractive pastime for most in the western world [8]. Recent data reported that 29% of adults’ alcohol consumption in the United Kingdom is hazardous [9], categorized as typically drinking over 14 units of alcohol a week. This remains to be a global problem, with 39.5% of drinkers reporting heavy episodic drinking globally [10].

Similar to alcohol consumption, disordered eating (or binge eating) has been normalized by society [11,12]. Binge eating is the consumption of a larger amount of food than what is expected of an individual, in a fixed period, typically accompanied by a sense of lack of control [13]. This behavior is typically used as a coping mechanism in response to adverse negative events or stimuli [14], which may be conscious or unconscious (thus, the term *emotional eating* is often used). Binge eating is linked to classified eating disorders, including binge eating disorder, bulimia nervosa, anorexia nervosa, and eating disorders not otherwise specified [14]. However, this act may also be present in subclinical populations without a diagnosis. Over the last decade, hospital admissions for both eating disorders and mental health disorders have risen [15,16]. Technology may have contributed to the increase in disordered eating due to the promotion of unhealthy behaviors (eg, restrictive dieting, which leads to binge eating [17,18] and mukbang trends [19]) on web-based platforms and the 24/7 access to this content. Advances in technology may have also contributed to the increased prevalence of gambling and problem gambling [20]. Due to the extensive growth in web-based gambling, the opportunity to gamble is no longer limited to physical gambling establishments.

Theoretical models and empirical evidence suggest that alcohol misuse, binge eating, and gambling may share similar psychological mechanisms of action [21]. For example, individuals with increased impulsivity engage in greater and more frequent health and financial risk behaviors, such as gambling, binge eating, and binge drinking [22]. Similarly, alcohol misuse, binge eating, and gambling have all been characterized as compulsive behaviors, with the initiation of each thought to be driven by relevant behavior-related and emotive cues [23,24], suggesting cue-controlled behavior. The identification of common underlying behavioral mechanisms suggests that interventions targeting these broad behaviors, rather than tailoring them to specific symptoms, may be beneficial for a greater number of individuals [25,26].

Many individuals actively avoid seeking a formal diagnosis of their alcohol misuse, binge eating, or gambling due to negative self and social stigma [27-29]. This is despite many individuals recognizing the negative impact of these addictions on their quality of life (including their physical health, mental distress, and financial burden [30,31]). Furthermore, individuals may not seek traditional face-to-face treatments due to geographical restrictions, availability, or time constraints [32]. This has resulted in a considerable treatment gap [33], with many people in need not seeking or receiving treatment.

eHealth interventions are able to bridge this treatment gap by allowing anonymity, overcoming location barriers, and providing *any-time* accessibility [32,34]. There have been several successful interventions for each behavior. Meta-analyses of alcohol, gambling, and binge eating behavior have concluded that eHealth interventions are efficacious, with a moderate-to-large effect size [35] found for binge eating [36] and moderate effect sizes for alcohol [37] and gambling [38]. Despite these findings, many web-based intervention assessments lack vigorous testing methods, such as randomized controlled trials (RCTs) to determine their behavior change effectiveness [39]. Alternatively, if the effectiveness of the web-based intervention is tested, the reasons for this effectiveness, such as the behavior change techniques (BCTs) used, may not be explored [40]. This can be observed in many recent meta-analyses. Instead of this top-down approach, interventions should be developed following a bottom-up approach and the values of the Experimental Medicine Approach [41,42]. This approach focuses on examining the core mechanisms that are behind a behavior and holds particular use in intervention development, placing emphasis on the importance of identifying and targeting the malleable psychological processes or surroundings of an individual for behavior change [42].

The BCT taxonomy provides an overview of methods for behavior change and their hierarchical structure [43]. By looking at the underpinning mechanisms behind a behavior, an appropriate BCT, or more likely, a combination of BCTs can be identified to target a behavior effectively. Using this theory-based procedure to design an intervention means that optimum results are achieved for both the intervention users and business owners due to increased user satisfaction. eHealth interventions are often favored by businesses because of their cost-effectiveness in comparison with traditional face-to-face services. This cost-effectiveness increases dramatically when the transdiagnostic capability of a web-based intervention—when one treatment is used to target multiple behaviors—is considered [44]. Web-based interventions are capable of being transdiagnostic, as their content is often open for interpretation, focusing on user reflection and self-monitoring of behavior.

Research has confirmed the efficacy of many eHealth interventions in changing addictive behaviors such as binge eating, gambling, and alcohol behavior [36-38]. Some potential moderators have been discussed. For example, gambling intervention effectiveness was found to be moderated by gender, with greater success in male participants [38]. In addition, group components of gambling interventions were found to have long-lasting behavioral effects [38]. In alcohol interventions, the delivery type was explored [37]. Greater success in reduced alcohol consumption was reported in interventions that provided supplementary alcohol-related materials, which were not *commercially available*, possibly implying the importance of in-depth, systematic intervention design for specific problems [37]. Motivation was also considered, with particular attention paid to the impact of eating intervention effectiveness on this, where over a quarter of studies used participants who were seeking weight loss through diet and exercise or bariatric surgery [36]. However, no specific consideration of BCTs were found in these studies. This is still common in several papers on behavior change [45,46]. This holds significant because of the concerns that many eHealth interventions are not developed on the basis of best practices in behavior change. Often, interventions are developed favoring intuition over research findings or those that claim to be evidence based are actually *evidence inspired* at best due to companies’ preconceived ideas [46,47]. This is detrimental not only to intervention users and those delivering them but also for future research in behavior change and intervention development, as it leads to studies reporting effectiveness in behavior change but not identifying the specific mechanism behind this. The same applies to many evidence-based papers, with it being common for journal articles to lack detail on an intervention to allow for BCT coding [45], which will limit our current understanding of BCTs and mechanisms of behavior. Although it is a relatively new concept, the BCT taxonomy [43] provides a common language among behavior change papers, allowing effective synthesis of evidence-based papers to enhance behavior change knowledge.

The use of this taxonomy has led to valuable findings. Conducting a review on a single behavior type allows knowledge to be summarized in this specific area. This allows BCTs to be identified as effective for this topic, which holds value because BCT effectiveness is dependent on context, such as the type of behavior targeted [47]. Existing research has highlighted promising BCTs for alcohol, gambling, and binge eating. For example, Crane et al [48] identified 12 BCTs that were deemed as promising for behavior change, including self-monitoring of behavior, goal setting (behavior), feedback on behavior, and behavior substitution. Similar studies have been conducted on gambling, highlighting feedback on behavior, goal setting, social comparison, and exposure as useful BCT techniques to reduce gambling behavior [49]. A recent meta-analysis of BCTs on healthy eating interventions, which aimed to reduce unhealthy eating behaviors such as binge eating, similarly identified self-monitoring of both behavior and outcomes of behavior as a critical BCT for behavior change effectiveness [50]. A synthesis on BCTs can also be performed on multiple behaviors to inform the development of transdiagnostic interventions. However, there are no existing reviews examining which BCTs may be effective for binge eating, gambling, and alcohol consumption together.

### Objectives

The aim of this systematic review is to (1) identify web-based interventions targeting a reduction in alcohol consumption, binge eating, and gambling behavior; we focused on these 3 behaviors due to their increasing prevalence in subclinical populations, similar underlying psychological mechanisms, and the widespread accessibility to materials for these behaviors; (2) determine the effectiveness of these interventions; and (3) identify the BCTs used in these interventions, examining the similarities in techniques to inform the development of a future transdiagnostic intervention.

## Methods

### Literature Search

**Figure 1:**
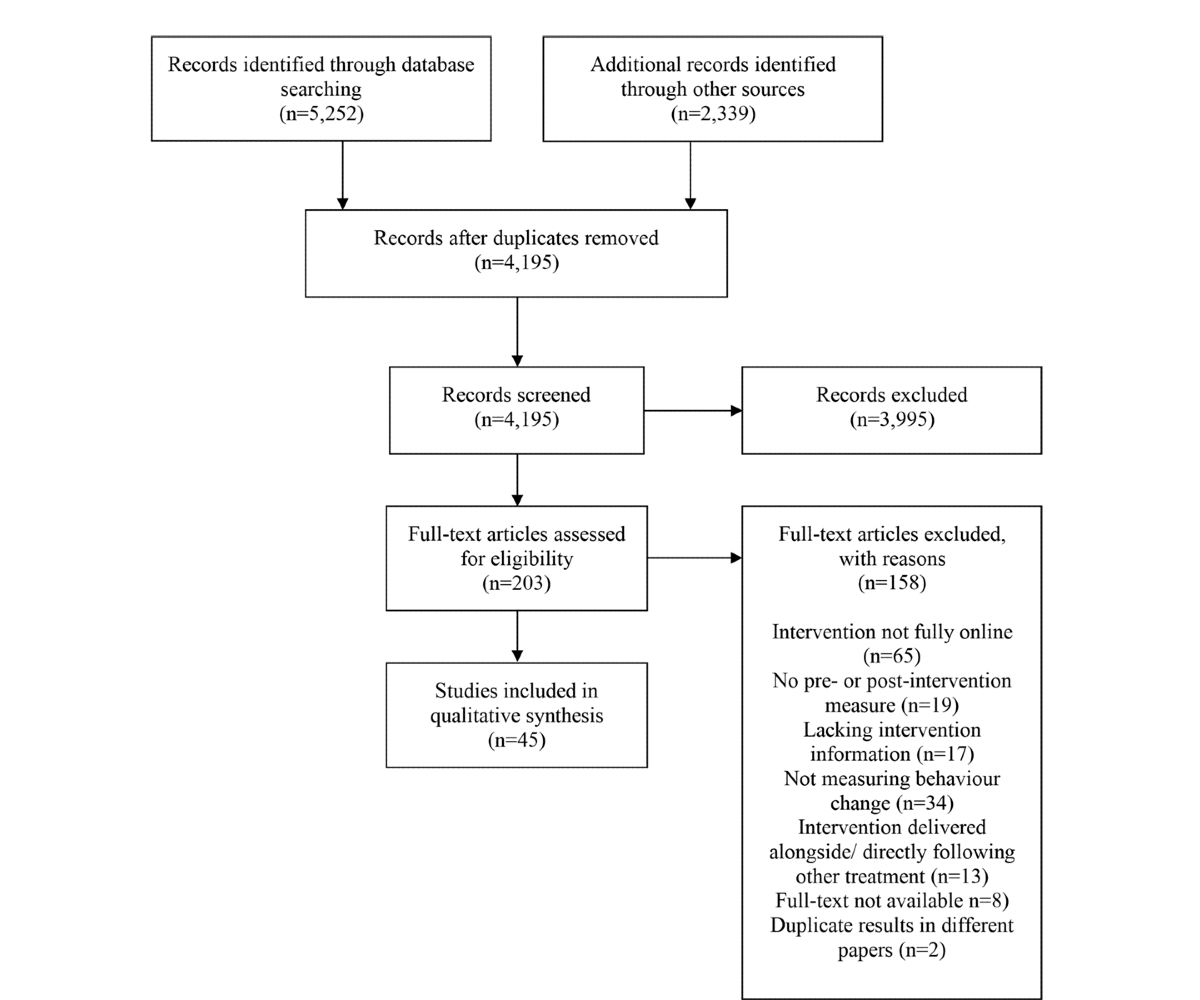
PRISMA (Preferred Reporting Items for Systematic Reviews and Meta-analyses) flowchart followed in this study.

Scoping searches were conducted in November 2019 using several electronic databases. Following these searches, we refined our search criteria and registered the protocol for this study on the Open Science Framework [51]. Full searches were conducted in December 2019 on PsycINFO, PubMed, and Scopus following the Preferred Reporting Items for Systematic Reviews and Meta-analyses (PRISMA) guidelines (Figure 1). Search terms can be found in Multimedia Appendix 1.

### Eligibility Criteria

Relevant articles were identified by a single author (GH). A second coder (RE) independently assessed the eligibility of the selected papers. There were no major disagreements between the authors. To be eligible for inclusion in the review, studies had to be either case controlled or RCTs that examined the effectiveness of a web-based intervention. Intervention effectiveness was determined as measured behavior change (eg, reduction in units of alcohol consumed) rather than user satisfaction. Eligible studies had to examine interventions to reduce alcohol use, binge eating, or gambling, although these outcomes did not have to be the primary outcome measure of the study. Other inclusion criteria were as follows: measurements reported at baseline and immediately postintervention for the relevant behavior change, *either* sufficient detail of the intervention content in the paper *or* a direct link to the study protocol to allow for BCTs to be coded, and research published in the past 20 years with a human-only sample. Exclusion criteria included interventions that were not entirely delivered on the web, which means that any intervention with a face-to-face element was not eligible. Interventions not delivered independently of other behavior change methods (eg, an eating intervention in individuals who had previously undergone a gastric bypass) and interventions that aimed to maintain, rather than change, behavior were not included. Similarly, interventions aimed at preventing the onset of problematic behavior were not included. However, if such interventions aimed to prevent the worsening of behavior and measured this behavior, rather than reported user intentions, these studies were eligible [52]. Data collected must have been from a validated measure (eg, Alcohol Use Disorder Identification Test [AUDIT-C]) [53] or self-reported retrospective recall of quantity or frequency of behaviors (eg, Alcohol Timeline FollowBack [54]). Measurements had to encapsulate behavior change rather than acceptability, feasibility, or user satisfaction.

### Data Extraction

Data were extracted from eligible studies by a single author (GH) using a data extraction form. Variables in the data extraction form included participant characteristics (including details of control groups), intervention characteristics, intervention effectiveness, and BCTs used in the intervention (Multimedia Appendices 2-4 [42,52,55-99]). BCTs were coded using the BCT taxonomy version 1 [43]. Some participants (eg, Crombie et al [55] and Pederson et al [56]) completed unrelated tasks as a comparator, referred to as an active control group. Owing to their inability to eliminate placebo effects [100], if an active control group was used and adequate information was provided, the authors coded both interventions for BCTs. If a no-treatment control group was used, typically in the form of a waitlist condition or assessment-only condition, the intervention was not coded. The coding of BCTs was completed independently by 2 authors (GH and HM), with any disagreements resolved. There were no major disagreements with a Cohen κ score of 0.83. The authors were contacted if relevant data were missing from eligible papers. After the BCTs were coded, frequency counts were performed to examine the prevalence of BCTs. Data extracted for eligible studies [52,57-98] can be located in Multimedia Appendices 2-4. The BCT Periodic Table by Armitage et al [99] was used for labeling BCTs, rather than the BCT taxonomy v1 numbers [43], for ease of reading.

### Quality Assessment

Risk of bias was assessed using the Office of Health Assessment and Translation (OHAT) Risk of Bias Rating Tool [101] selected due to its use in health-related studies and RCTs. The areas considered were as follows: condition randomization and concealment (where applicable), the appropriateness of control groups (if used), attrition rates, reporting bias, and the reliability and validity of measures. Item 5 from the OHAT tool was removed as it was only applicable to animal experiments, which were not included in this review. The number of items completed was dependent on study design, with a total score of either 30 (RCTs) or 18 (case controlled). Questions were answered using a 4-point scale ranging from *definitely low risk of bias* to *definitely high risk of bias*. Direct evidence was required for the scoring of definite high or low bias. Probable risk was scored if evidence was missing or indirect. A score of 3 coincided with a *definitely low risk of bias* and zero with a *definitely high risk of bias*. High quality was indicated by a score of over 70%, and medium quality was indicated by a score over 50% [101]. Despite the recommendation of 70%, the existing literature has also used 80% as a high-quality threshold. Results using both thresholds were compared.

### Data Synthesis

An overall frequency count of BCTs used in the interventions was performed. BCTs were also counted when ineffective and when low-quality studies (<70% OHAT score and <80% OHAT score) were excluded, with results compared. The effectiveness of an intervention was determined by a statistically significant effect (*P*<.05) on behavior change in drinking, gambling, or eating. If a paper reported mixed findings regarding behavior change effectiveness, we used the most relevant variable as an indicator of effectiveness. The variable tended to be the overall effect reported or the most relevant measure of alcohol, binge eating, or gambling behavior if the intervention’s primary aims were not directly to promote this change. For example, an intervention that aimed to promote weight loss by targeting binge eating was eligible (Lyzwinski et al [95]). Similarly, an intervention with the primary aim to reduce drunk driving was eligible as it also targeted alcohol consumption (Bingham et al [62]). Studies that reported a statistically significant reduction in the relevant behavior change measure were therefore included in the frequency count of *effective interventions*. Similarly, those scoring over 70% on the OHAT risk of bias tool were included in the frequency count of *high-quality papers*. Finally, those that reported both a statistically significant effect in the relevant behavior change measure and a score of over 70% on the OHAT risk of bias tool were included in the *effective and high-quality papers*. The 5 most commonly used BCTs were reported for each study (Table 1); if BCT frequencies were equal, over 5 were reported in the results.

**Table 1:** A summary table of the behavior change techniques identified in frequency counts.

Frequency count key	BCT^a^ item						
	PS^b^	FOB^c^	SB^d^	SOB^e^	IPB^f^	ISEC^g^	SC^h^
Total^i^	✓^j^	✓	✓	—^k^	✓	✓	✓
Effective^l^	—	✓	✓	✓	✓	—	✓
>70% OHAT^m^	✓	✓	✓	—	✓	—	✓
>80% OHAT^n^	—	✓	✓	✓	✓	—	✓
>70% and effective^o^	—	✓	✓	✓	✓	—	✓

^a^BCT: behavior change technique.

^b^PS: problem solving.

^c^FOB: feedback on behavior.

^d^SB: self-monitoring of behavior*.*

^e^SOB: self-monitoring of outcomes of behavior.

^f^IPB: instruction on how to perform the behavior.

^g^ISEC: information about social and environmental consequences.

^h^SC: social comparison.

^i^Total: all eligible papers.

^j^✓: Presence of behavior change technique.

^k^—: Absence of behavior change technique.

^l^Effective: all papers that concluded a significant reduction in the specific target behavior measure from pre to postintervention.

^m^All papers deemed as high quality, which scored over 70% on the Office of Health Assessment and Translation (OHAT) risk of bias tool.

^n^All papers that scored over 80% on the OHAT risk of bias tool.

^o^All papers that scored over 70% on the OHAT risk of bias tool and were deemed as effective in target behavior reduction.

## Results

### Searches and Study Information

The search yielded a total of 5252 papers: 2672 alcohol papers, 1670 gambling papers, and 910 binge eating papers. After duplicates were removed, 4152 articles remained. Papers were then screened via title, abstract, and full text. Of those eligible papers, references were also examined (n=2339). This screening identified 2 papers that were eligible for the study. In total, 45 studies were identified as eligible for this review: 32 studies (71%) describing interventions for alcohol use, 7 (16%) describing interventions for gambling, and 6 (13%) describing interventions for binge eating.

Most eligible papers (37/45; 82%) were RCTs with a waitlist control group. The mean duration of intervention was 6 weeks. Interventions ranged from less than 1 hour to 1 year. A computer was the most frequently used intervention device. Participants were typically from at-risk groups or displayed concerning behavior (Multimedia Appendices 2-4). Pre- and postintervention behavior was most commonly assessed using the AUDIT-C for alcohol, the South Oaks Gambling Screen for gambling, and the Eating Disorder Examination Questionnaire for binge eating.

The most commonly used BCT across all 45 papers was self-monitoring of behavior (BCT item 2.3), which was present in 39/45 (87%) of interventions. Feedback on behavior (BCT item 2.2; 30/45, 67%), social comparison (BCT item 6.2; 27/45, 60%), information about social and environmental consequences (BCT item 5.3; 26/45, 58%), instruction on how to perform a behavior (BCT item 4.1; 25/45, 56%), and problem solving (BCT item 1.2; 25/45, 56%) were other commonly used techniques. Data on BCT frequency counts can be found in Multimedia Appendices 2-4.

### Effective Interventions

In total, 66% (21/32) of alcohol interventions, 83% (5/6) of eating behavior interventions, and 43% (3/7) of gambling interventions were effective. After excluding unsuccessful interventions, 64% (29/45) of the eligible interventions remained. In total, 21 interventions targeted alcohol behavior (72%), 5 targeted binge eating (17%), and 3 targeted gambling (10%). A frequency count of effective-only papers revealed that the most commonly used BCTs remained self-monitoring of behavior (BCT item 2.3), feedback on behavior (BCT item 2.2), social comparison (BCT item 6.2), and instruction on how to perform a behavior (4.1). Information about social and environmental consequences (BCT item 5.3) was replaced by BCT item 2.4 (self-monitoring of outcomes of behavior). Thus, the BCTs used in effective studies were the same as those used most frequently across all studies.

### High-Quality Papers

In total, 56% (25/45) of papers were rated as having high quality (OHAT>70%). Of these 25 papers, 18 (72%) papers targeted alcohol misuse, 4 (16%) targeted gambling, and 3 (11%) targeted binge eating. When examining only high-quality studies, the most frequent BCTs used were self-monitoring of behavior (BCT item 2.3), instruction on how to perform a behavior (BCT item 4.1), feedback on behavior (BCT item 2.2), and social comparison (BCT item 6.2). Problem solving (BCT item 1.2) was also identified as a commonly used BCT in high-quality papers.

When the threshold for a high-quality study was increased (OHAT>80%), 9 papers retained this classification: 6 targeting alcohol abuse and 3 targeting gambling. No binge eating studies met this criterion. Self-monitoring of behavior (BCT item 2.3), feedback on behavior (BCT item 2.2), social comparison (BCT item 6.2), and instruction on how to perform a behavior (BCT item 4.1) remained to be the most commonly used BCTs. Problem solving (BCT item 1.2) was no longer within the top 5 most frequently used BCTs. Self-monitoring of outcomes of behavior replaced problem solving (BCT item 2.4), meaning results were consistent with the effective-only frequency count.

### Effective and High-Quality Papers

In total, 16 of 45 papers (36%) were classified as being effective and of high quality (>70% OHAT score). Of these, 10 (63%) focused on alcohol misuse, 3 (19%) on binge eating, and 3 (19%) on gambling. The most commonly used BCTs in these papers were self-monitoring of behavior (BCT item 2.3), feedback on behavior (BCT item 2.2), self-monitoring of outcomes of behavior (BCT item 2.4), instruction on how to perform a behavior (BCT item 4.1), and social comparison (BCT item 6.2). This is consistent with the findings of the effective-only frequency count and the OHAT score over an 80% frequency count.

### Common BCTs Among Frequency Counts

In total, 7 BCTs were identified as commonly used within the frequency counts. These were problem solving (BCT item 1.2), feedback on behavior (BCT item 2.2), self-monitoring of behavior (BCT item 2.3), self-monitoring of outcomes of behavior (BCT item 2.4), instruction on how to perform a behavior (BCT item 4.1), information about social and environmental consequences (BCT item 5.3), and social comparison (BCT item 6.2). Within the 5 frequency counts performed, the techniques feedback on behavior (BCT item 2.2), self-monitoring of behavior (BCT item 2.3), instruction on how to perform a behavior (BCT item 4.1), and social comparison (BCT item 6.2) were present in all counts. Self-monitoring of outcomes of behavior (BCT item 2.4) was identified in the majority of frequency counts (3). Problem solving (BCT item 1.2) was present in 2 frequency counts, and information about social and environmental consequences (BCT item 5.3) was present in 1 frequency count.

## Discussion

### Principal Findings

This systematic review identified 7 commonly used BCTs in web-based alcohol, gambling, and binge eating eHealth interventions: problem solving, feedback on behavior, self-monitoring of behavior, self-monitoring of outcomes of behavior, instruction on how to perform a behavior, information about social and environmental consequences, and social comparison. Although the most frequent BCTs used in papers varied when intervention effectiveness and study quality were taken into account, this variation was minor, with feedback on behavior, self-monitoring of outcomes, instruction on how to perform a behavior, and social comparison present in all frequency counts. This suggests that researchers testing interventions for these behaviors use similar approaches. Self-monitoring of outcomes was present in 3 out of 5 frequency counts, problem solving was present in 2 frequency counts, and information about social and environmental consequences was present in 1 frequency count. Attention should be paid to items feedback on behavior, self-monitoring of behavior, self-monitoring of outcomes, instruction on how to perform a behavior, and social comparison, which were the frequency count results for both high-quality and effective studies. Most papers were of high quality and were RCTs. However, when looking at the breakdown of studies in each behavior type, these were very imbalanced, with 71% targeting alcohol consumption, 16% gambling, and 13% binge eating. Study effectiveness varied within each behavior type, meaning that the studies included in effective frequency counts were not equally split (72% alcohol, 17% binge eating, and 10% gambling). However, this was expected due to the prevalence of interest in alcohol research, and thus, more published studies in this area. Despite highlighting the results from the frequency count on high-quality and effective papers, BCTs identified in the other frequency counts are relevant because this presence is likely to reflect the feasibility, acceptability, or cost-effectiveness of a technique, aspects of an intervention that hold great importance.

These 7 BCTs should be focused on when designing web-based interventions in these addictive areas. It can be presumed that these BCTs were included because of their relevance to addictive behavior change, rather than purely from theoretical findings, as many interventions do not prioritize this theory and follow a systematic procedure when designing programs [102]. For example, self-monitoring of behavior (BCT item 2.3) is an important step in interventions to raise a user’s awareness of their behavior and was identified in 86% of eligible papers. Similarly, providing instructions on how to perform a behavior (BCT item 4.1) was present in 55% of eligible papers. This is something that should be included in interventions that aim to provoke behavior change [103]. This means that the findings of this paper will be easily transferrable to the practical development of interventions, prompting to prioritize areas that should already have been considered by developers rather than causing web-based interventions to be completely redeveloped.

These results are consistent with other findings. For example, Michie et al [50] highlighted the role of the BCT self-monitoring of both behavior and outcomes of behavior in a healthy eating intervention. Both self-monitoring of behavior and self-monitoring of behavior outcomes were identified in the frequency counts of this review, with self-monitoring of behavior present in all frequency counts and self-monitoring of outcomes of behavior present in 3 of the 5 counts. The methodology of this study differed, with a meta-regression conducted by Michie et al [50] to examine the effectiveness of specific BCTs. However, we did not conduct a meta-analysis due to the heterogeneity of studies, and the aim of this paper is to identify few BCTs to inform further development interventions, rather than focusing primarily on examining effectiveness and grouping BCTs together.

In a content analysis of alcohol reduction apps, Crane et al [48] identified 12 promising BCTs for effective behavior change. Similar to this study, self-monitoring of behavior, feedback on behavior, information about consequences, and social comparison were identified by the authors as useful BCTs. Instruction on how to perform the behavior, self-monitoring of behavior, and problem solving, which were identified in this study, were not identified as promising by Crane et al [48]. A similar level of support was found for the review by Rodda et al [49], with feedback on behavior, social comparison, and self-monitoring of behavior and outcomes of behavior identified as useful BCTs for behavior change in both this study and the review by Rodda et al [49]. Other BCTs were identified by authors, which this study did not support, for example, exposure and behavior substitution. However, inconsistent findings are to be expected in BCT research, as these techniques are highly dependent on target behavior, which were not identical.

### Limitations

The effectiveness of BCTs is dependent on the behavior targeted by an intervention. This means that the results are only generalizable to these specific behaviors rather than behavior change on a wider scale. Despite this, it is encouraging that there were clearly common BCTs across the 3 identified problem behaviors, which can provide useful guidance for future intervention development. In addition to the BCTs used and the intervention’s topic, multiple other variables contribute to the behavior change success of an intervention. These include motivation, opportunity, and perceived capability to change, which simultaneously act on behavior change and can be referred to as the COM-B (Capability, Opportunity, Motivation–Behavior) model [102]. The Behavior Change Wheel combines these behavioral sources with intervention functions, including education, persuasion and environmental restructuring, and policy categories such as guidelines and regulations, to provide an in-depth framework of behavior change [102]. Future research should take into account the interactions between these motivational and circumstantial variables and the mechanisms of behavior change. Without this consideration of factors including help seeking and resource availability, it is an oversimplification of behavior change theory and may lead to considerable resources being wasted on poorly implemented interventions. Therefore, this review can only provide guidance for intervention frameworks, rather than recommending a concrete intervention structure.

Although it provides an effective overview of BCTs, details in BCT taxonomy v1 are sometimes lacking and relatively subjective [104]. For example, BCT item 2.2 refers to feedback on behavior. This includes all possible feedback, with no specification on feedback type or delivery. Similarly, BCT item 5.1 is coded for any information about health consequences. Research has shown that the extremity of information content impacts one’s behavioral response [105,106], which in turn will determine an intervention’s behavior change effectiveness. This idea can be applied to the majority of the 93 BCTs identified within the taxonomy. In addition, there are other taxonomies, such as the Self-Enactable Techniques [107], which identify other techniques that may be relevant in behavior change interventions and are not directly listed in BCT taxonomy. For example, distraction (item 63), reflecting on the ability to perform behavior (item 99), and normalized behavior (item 114).

Finally, the scope of this paper was limited by the evidence base, which is biased toward positive, statistically significant publications [108]. It is possible that there are studies on behavior change interventions that used these BCTs but were not published due to the intervention finding no significant results [109]. Gray literature was not included in this review. The inclusion of this information is currently under debate, with a lack of consensus on how to conduct a systematic review of BCTs most effectively [99]. The exclusion of gray literature may limit the scope of a review, whereas inclusion may be able to bridge the research gap from publication bias as well as the time delay between conducting and publishing a study. However, including gray literature in a systematic review can lead to difficulties in data extraction and synthesis due to the lack of publishing standards being followed by authors [109]. This means that researcher bias is likely within data interpretation, justifying our exclusion of these sources. Furthermore, this review is limited by the interventions that exist and what is achievable with technology at the current time. In the future, it is expected that interventions will have more advanced features, which may lead to different BCT recommendations.

### Implications

These results can inform the development of new web-based addiction treatments, providing recommendations of BCTs shown to be effective in past research to form the core of alcohol, binge eating, and gambling programs. This paper also highlights the importance of using evidence-based theory while developing behavior change interventions, which many treatments lack. Theory-based interventions are not only more likely to result in effective behavior change [110] but also allow a richer evaluation of interventions, enabling one to identify the active components of an intervention [102]. Ultimately, the consideration of behavior change theory and the testing of BCT effectiveness during the early stages of design means that addiction-based interventions as well as interventions targeting wider behaviors will result in greater, long-lasting behavior change [104]. Highlighted in the Behavior Change Wheel [102], behavior change is the result of an interacting system of individual and societal factors. Future research must adopt the experimental medicine approach [41,42] and examine not only whether these BCTs are present in interventions but also if they are the active mechanism of behavior change.

### Conclusions

Alcohol consumption, binge eating behavior, and gambling can all be classified as compulsive or impulsive due to the neural mechanisms of reward that they impact. Owing to this comorbidity, transdiagnostic interventions may be used as a potential treatment. This systematic review identified the BCTs present in 42 web-based interventions that targeted one of these 3 behaviors. The authors explored the commonalities between BCTs identified, controlling studies for quality and effectiveness, which can inform the development of future transdiagnostic interventions.

## Appendix

Multimedia Appendix 1 Search terms and individual frequency counts.

Multimedia Appendix 2 Study characteristics for eligible studies that targeted alcohol consumption.

Multimedia Appendix 3 Study characteristics for eligible studies that targeted gambling.

Multimedia Appendix 4 Study characteristics for eligible studies that targeted binge eating.

## Abbreviations

BCT: behavior change technique
OHAT: Office of Health Assessment and Translation
RCT: randomized controlled trial
