# Supplementary material for: Identification of Behavior Change Techniques From Successful Web-Based Interventions Targeting Alcohol Consumption, Binge Eating, and Gambling: Systematic Review
Source: J Med Internet Res. 2021 Feb 9;23(2):e22694. doi: 10.2196/22694 (PMC7902193; doi:10.2196/22694)
Supplement: Multimedia Appendix 1 [file jmir_v23i2e22694_app1.docx]

1

(Online OR web* OR internet OR mobile* OR smartphone* OR e-health OR m-health OR “electronic health” OR telemedicine) AND (intervention* OR program* OR technique* OR “behavio* change*” OR bct OR treatment* OR pathway* OR therap*) AND (alcohol OR ethanol AND hazardous OR harmful OR bing*)

2

(Online OR web* OR internet OR mobile* OR smartphone* OR e-health OR m-health OR “electronic health” OR telemedicine) AND (intervention* OR program* OR technique* OR “behavio* change*” OR bct OR treatment* OR pathway* OR therap*) AND (“emotional eating” OR “binge eating” OR overeating)

3

(Online OR web* OR internet OR mobile* OR smartphone* OR e-health OR m-health OR “electronic health” OR telemedicine) AND (intervention* OR program* OR technique* OR “behavio* change*” OR bct OR treatment* OR pathway* OR therap*) AND (gambling OR gamble)

| *Table 1:* Frequency count totals. | | | | | | | | |
| --- | --- | --- | --- | --- | --- | --- | --- | --- |
| **BCT** | **Alcohol** | **Gambling** | **Binge eating** | **Total** | **Effective only** | **>70% OHAT** | **>80% OHAT** | **>70% and effective** |
| **GSB** | 13 | 2 | 0 | 15 | 5 | 11 | 4 | 5 |
| **PS** | 18 | 2 | **4** | **24** | 13 | **17** | 4 | 7 |
| **AP** | 10 | 0 | 1 | 11 | 4 | 7 | 1 | 2 |
| **RBG** | 3 | 0 | 0 | 3 | 1 | 1 | 1 | 0 |
| **C** | 2 | 1 | 0 | 3 | 2 | 2 | 1 | 0 |
| **FOB** | **21** | **5** | **3** | **29** | **20** | **16** | **6** | **12** |
| **SB** | **24** | **4** | **5** | **33** | **23** | **21** | **7** | **14** |
| **SOB** | 19 | 1 | **3** | 23 | **19** | 12 | **5** | **11** |
| **B** | 7 | 0 | 0 | 7 | 5 | 2 | 1 | 0 |
| **FOO** | 8 | 1 | 1 | 10 | 9 | 4 | 0 | 4 |
| **SSU** | 2 | 0 | 2 | 4 | 2 | 2 | 0 | 1 |
| **IPB** | 19 | **3** | 2 | **24** | **16** | **17** | **5** | **10** |
| **IAA** | 9 | 2 | **3** | 14 | 8 | 12 | 2 | 5 |
| **R** | 0 | 1 | 0 | 1 | 0 | 1 | 0 | 0 |
| **BE** | 0 | 0 | 1 | 1 | 1 | 0 | 0 | 0 |
| **IHC** | **21** | 1 | 0 | 22 | 14 | 12 | 4 | 7 |
| **ISEC** | **23** | 2 | 1 | **26** | 14 | 13 | 4 | 6 |
| **MEC** | 3 | 0 | 2 | 5 | 5 | 4 | 1 | 3 |
| **IEC** | 1 | 1 | 0 | 2 | 1 | 2 | 2 | 1 |
| **DOB** | 0 | 0 | 1 | 1 | 1 | 1 | 0 | 1 |
| **SC** | **25** | 2 | 2 | **29** | **19** | **15** | **6** | **9** |
| **PC** | 1 | 0 | 0 | 1 | 0 | 1 | 1 | 0 |
| **RAS** | 1 | 0 | 0 | 1 | 1 | 1 | 0 | 1 |
| **E** | 0 | 1 | 1 | 2 | 2 | 2 | 1 | 2 |
| **BPR** | 2 | 0 | 2 | 4 | 2 | 3 | 0 | 1 |
| **BS** | 6 | 1 | **5** | 12 | 9 | 8 | 3 | 5 |
| **HR** | 0 | 0 | 1 | 1 | 1 | 1 | 0 | 1 |
| **O** | 0 | 0 | 1 | 1 | 1 | 0 | 0 | 0 |
| **GT** | 0 | 0 | 1 | 1 | 1 | 1 | 0 | 1 |
| **CS** | 0 | 2 | 1 | 3 | 3 | 1 | 0 | 1 |
| **PAC** | 9 | 0 | 1 | 10 | 6 | 5 | 1 | 2 |
| **CIFO** | 0 | 0 | 2 | 2 | 2 | 2 | 0 | 1 |
| **MRB** | 1 | 0 | 0 | 1 | 1 | 1 | 0 | 1 |
| **RNE** | 3 | 2 | 2 | 7 | 5 | 7 | 2 | 4 |
| **RPE** | 1 | 1 | 0 | 2 | 1 | 0 | 0 | 0 |
| **AEB** | 2 | 0 | 1 | 3 | 2 | 1 | 1 | 0 |
| **F/R** | 1 | 0 | 1 | 2 | 2 | 2 | 2 | 0 |
| **IB** | 4 | 2 | 0 | 6 | 5 | 3 | 3 | 4 |
| **VSI** | 0 | 0 | 2 | 2 | 2 | 1 | 0 | 0 |
