# Supplementary material for: Identification of Behavior Change Techniques From Successful Web-Based Interventions Targeting Alcohol Consumption, Binge Eating, and Gambling: Systematic Review
Source: J Med Internet Res. 2021 Feb 9;23(2):e22694. doi: 10.2196/22694 (PMC7902193; doi:10.2196/22694)
Supplement: Multimedia Appendix 2 [file jmir_v23i2e22694_app2.docx]

Multimedia Appendix 2. Study characteristics for eligible studies which targeted Alcohol consumption.

| Authors | Participants | Intervention and control | BCTs | Outcome measures | Notes | Results | Risk of bias |
| --- | --- | --- | --- | --- | --- | --- | --- |
| Alcohol | | | | | | | |
| Andrade et al. (2016). | N= 929.  Mean age: 40.  Country: Brazil.  Inclusion criteria: Created an online account in allocated timeframe.  Exclusion criteria: None. | Name: Bebermenos.  Device: Computer.  Duration: 6 weeks.  Aim: To reduce alcohol consumption and alcohol-related problems and examine the impact of dependency level.  Control: No control group. | N= 8.  GSB, AP, RBG, FOB, SB, SOB, ISEC, PAC. | TLFB, AUDIT, Readiness to change subscale of the Recovery Community Organisation (RCO). | Authors of paper affiliated with the e-Health project. | Intervention significantly reduced alcohol consumption in high-risk drinkers and dependent drinker groups (p=.02). No differences were found between groups. | 67%,  12/18. |
| Arnaud et al. (2016). | N= 211.  Mean age: 17.  Country: Western Europe.  Inclusion criteria: Aged 16 to 18, had internet access, from Sweden, Czech Republic, Germany or Belgium, in an at-risk substance use group.  Exclusion criteria: None. | Name: WISEteens.  Device: Computer.  Duration: Up to 30 minutes.  Aim: To reduce substance use in at-risk adolescents.  Control: Waiting list condition. | N= 8.  PS, FOB, B, FOO, IAA, IHC, SC, PAC. | AUDIT-C, intentions to reduce drinking Likert-scale item. |  | Intervention group showed significantly greater reduction in AUDIT scores than the control (p=.01). However, when EMI data was used to overcome high attrition rates, this was non-significant. | 60%,  18/30. |
| Bertholet et al. (2015). | N= 737.  Mean age: 21.  Inclusion criteria:  Country: Switzerland.  Male, 21 years old, reported unhealthy alcohol use (>14 drinks per week or >6 drinks per occasion in the past month), registered to C-SURF cohort.  Exclusion criteria: None. | Name: Alcooquizz.  Device: Computer.  Duration: Time reading feedback was not controlled.  Aim: To reduce unhealthy alcohol use in young, swiss men.  Control: Completed assessments only. | N= 5.  FOB, B, FOO, IHC, SC. | TLFB, AUDIT, alcohol related consequences from Weschler et al. |  | Intervention led to a significant decrease in drinking frequency and AUDIT scores. However, there was no significant reduction in binge drinking behaviour or overall intervention effect over time. | 63%,  19/30. |
| Bertholet et al. (2017). | N= 112.  Mean age: 33.  Country: Switzerland and Canada.  Inclusion criteria: Over 18 years old, reported unhealthy alcohol use (>14 drinks per week or >1 binge episode per month).  Exclusion criteria: None. | Name: Alcooquizz.  Device: Mobile phone.  Duration: 5 modules with no time controls.  Aim: To reduce drinking behaviour in those with unhealthy alcohol use.  Control: No control group. | N= 8.  GSB, PS, FOB, SB, SOB, IHC, SC, MRB. | AUDIT, TLFB, Welschler’s measure of alcohol-related consequences. |  | Intervention significantly decreased drinking frequency (p<.01), binge frequency (p<.001) and typical number of drinks consumed per week (p<.01). App use significantly predicted lower alcohol consumption, but not frequency of drinking or binging. | 72%,  12/18. |
| Bewick et al. (2010). | N= 1112  Mean age: 22  Country: UK  Inclusion criteria: One of four pre-selected universities.  Exclusion criteria: None. | Name: Unitcheck  Device: Computer  Duration: 15 weeks.  Aim: To reduce drinking behaviour in student drinkers.  Control: Assessment only. | N= 8.  PS, SB, SOB, IPB, IHC, ISEC, SC, CS. | AUDIT, TLFB, Readiness to change survey (self-designed). |  | A longitudinal regression showed a significant intervention effect on drinking behaviour of participants (p<.01). The model showed an additional effect on drinking behaviour from repeated access to the intervention (p<.001). | 53%, 16/30. |
| Bewick et al. (2013). | N= 1618  Mean age: 21.  Country: UK.  Inclusion criteria: Current student at the University of Leeds.  Exclusion criteria: Self-reported non-drinkers not included in study. | Name: Unitcheck  Device: Computer  Duration: 15 weeks.  Aim: To reduce drinking behaviour in student drinkers.  Control: Assessment only. | N= 8.  PS, SB, SOB, IPB, IHC, ISEC, SC, CS. | CAGE, TLFB for drinking frequency and drinks consumed per occasion. |  | A longitudinal regression showed a significant intervention effect on drinking behaviour (p<.001). The model showed an additional effect on drinking behaviour from repeated access to the intervention (p<.001). | 57%, 17/30. |
| Bingham et al. (2011). | N= 742.  Mean age: Missing.  Country: US.  Inclusion criteria: Aged 18 to 20, first year Michigan university students, lived in a dormitory, no children.  Exclusion criteria: Non-traditional students. | Name: M-PASS (Michigan Prevention and Alcohol Safety for Students).  Device: Computer.  Duration: 9 weeks, 15 minutes per week.  Aim: To prevent risky drinking behaviour (mainly drink driving) in users.  Control: Waiting list control. | N= 10.  GSB, PS, FOB, IPB, IAA, ISEC, SC, BS, PAC, IB. | TLFB, one item from DDQ assessing frequency of drinking occasions, Readiness to Change Questionnaire (RTCQ), Young Adults Alcohol Problems Screening Test. | Research funded by NIAAA. | Intervention led to a significant reduction in quantity and frequency of drinking in women (p=.021), and binge drinking in both men (p=.045) and women (p<.001). No overall intervention effects were reported. | 57%,  17/30. |
| Crombie et al. (2018). | N= 707.  Mean age: 35.  Country: UK.  Inclusion criteria: From one of four selected disadvantaged areas in Scotland, reported two or more binge drinking episodes in the past month, ages 25 to 45.  Exclusion criteria: Enrolled in other alcohol treatment. | Name: TRAM (Texting to Reduce Alcohol Misuse).  Device: Mobile phone.  Duration: 112 text messages sent over 12 weeks.  Aim: To reduce binge drinking in disadvantaged men.  Control: Received 89 texts on general health, with no mention of alcohol. | N= 5.  GSB, PS, AP, IHC, ISEC. | TLFB. |  | No significant intervention effects were found. Despite this, intervention acceptability was high (96.4% would recommend intervention). | 77%,  22/30. |
| Deady et al. (2016). | N= 104.  Mean age: 22.  Country: Australia.  Inclusion criteria: Aged 18 to 25 in Australia, reported a score of over 8 on AUDIT and reported moderate depression (>7 on DAS-21).  Exclusion criteria: Displayed signs of suicidal ideations, reported weekly use of amphetamines or daily use of cannabis. | Name: DEAL project.  Device: Computer.  Duration: 4 weeks.  Aim: To reduce problematic alcohol use and depressive symptoms.  Control: Completed HealthWatch which focussed on exercise and mental health. | N= 7.  GSB, PB, AP, SB, SOB, MEC, RNE. | TOT-AL Alcohol Consumption measure, Patient Health Questionnaire Depression Test (PHQ-9). | Prior to testing, the main author has been involved in intervention development. | Intervention led to a significant decrease in drinks per week (p<.001). This was significantly fewer than the control group (p=.05). Intervention group reported a two-fold greater drinks per week reduction than the control group (p=.02). | 83%,  25/30. |
| Doumas and Hausveit (2008). | N= 52.  Mean age: 18.  Country: US.  Inclusion criteria: First year university students, enrolled in an athletic group.  Exclusion criteria: None. | Name: CheckYourDrink.  Device: Computer.  Duration: 15 minutes for assessment. Time with feedback was not limited.  Aim: To reduce overall drinking in first year athletic students.  Control: Web-based education group. | N= 8.  FOB, SB, SOB, FOO, IPB, IHC, ISEC, SC. | TLFB using NIAAA’s definitions of drinking, DDQ. |  | Intervention significantly reduced frequency of overall drinking, drinking to intoxication and maximum alcohol consumption on one occasion (p<.05). The greater one’s drinking risk, the greater the interventions effects (p=.03). | 73%,  22/30. |
| Doumas et al. (2011). | N= 350.  Mean age: 18.  Country: US.  Inclusion criteria: First year undergraduate students, aged 17 to 19.  Exclusion criteria: None. | Name: e-CHUG.  Device: Computer.  Duration: 30 minutes for assessment. Time reading feedback was not limited.  Aim: To decrease heavy drinking in first year students.  Control: Assessment only. | N= 6.  FOB, FOO, IPB, IHC, ISEC, SC. | TLFB, RAPI. |  | Intervention significantly reduced drinking to intoxication (p=.02) and maximum drinks on one occasion (p<.001) scores but did not significantly reduce weekly drinking quantity. The intervention effects were greater in higher-risk drinkers. | 57%,  17/30. |
| Dulin, Gonzalez and Campbell (2014). | N= 28.  Mean age: 34.  Country: Alaska.  Inclusion criteria: Aged 22 to 45, met DSM criteria for Alcohol Use Disorder, motivated to change behaviour.  Exclusion criteria: Enrolled in other substance abuse treatment, showed severe alcohol dependence (>30 on SAD-Q), met DSM criteria for bipolar disorder or psychosis. | Name: LBMI-A (Location-based monitoring and intervention for alcohol use disorders).  Device: Mobile phone.  Duration: 6 weeks, one weekly 15-minute session.  Aim: To reduce drinking and craving, help users manage problems and identify high-risk situations.  Control: No control group. | N= 12.  PS, FOB, SB, SOB, SSU, IAA, ISEC, MEC, BS, PAC, RPE, AEB. | TLFB. | Research funded by the NIAAA. | Intervention significantly reduced percentage of binge drinking days (p<.001), mean number of drinks (p<.001) and drinks per occasion (p<.01). Actively completing tasks was associated with a decrease in drinking behaviour (p<.05). | 67%,  12/18. |
| Fazzino, Rose and Helzer (2016). | N= 371.  Mean age: 20  Country: US and Canada.  Inclusion criteria: Undergraduate students, aged 18 to 26, reported drinking over 4/5 drinks in one sitting during the last month.  Exclusion criteria: None. | Name: e-CHUG (Electronic Check Up And Go).  Device: Computer or mobile phone on campus.  Duration: 20 minutes for assessment. Time with feedback was not controlled.  Aim: To investigate differences in alcohol consumption when using assessments alongside an online intervention.  Control: Assessment only. | N= 8.  FOB, SB, SOB, FOO, IPB, IHC, ISEC, SC. | TLFB, RRQ, Rutgers Alcohol Problem Index (RAPI), Protective Behavioural Strategies (PBS) scale, Blood Alcohol Concentration. |  | No significant decreases in the intervention group’s drink consumption or blood alcohol concentration. Intervention type did not significantly predict alcohol outcomes. | 73%,  22/30. |
| Ganz et al. (2018). | N= 346.  Mean age: 23.  Country: Germany.  Inclusion criteria: South-German university students.  Exclusion criteria: None. | Name: e-CHUG.  Device: Computer.  Duration: 30 minutes.  Aim: To reduce hazardous alcohol consumption in German students.  Control: Assessment only. | N= 8.  FOB, SB, SOB, FOO, IPB, IHC, ISEC, SC. | AUDIT-C, DDQ, Blood alcohol concentration, RAPI. | Had statistical power at baseline, however the sample size no longer met requirements at follow-up due to high attrition. | Intervention showed a significant decrease in reported number of drinks consumed over time (p<.001). No significant intervention effects were found on frequency of heavy drinking episodes or alcohol-related problems. | 70%,  21/30. |
| Haug et al. (2013). | N= 364  Mean age: 18.  Country: Switzerland.  Inclusion criteria: Pupils from selected schools in Zurich, owned a mobile phone.  Exclusion criteria: None. | Name: Alk-Check.  Device: Mobile phone.  Duration: 12 weeks.  Aim: To reduce frequency of risky single-occasion drinking (RSOD) and drinks consumed over the last month.  Control: No control group. | N= 10.  PS, FOB, SB, SOB, IPB, IHC, ISEC, SC, BS, PAC. | RSOD variable from the Daily Drinking Questionnaire (DDQ), TLFB. |  | Found a significant decrease in participants with less than 1 (p<.001) and less than 2 RSODs (p=.002) and the percentage of participants with alcohol-related problems in the last month (p=.009). Number of drinks in one occasion decreased, although this was non-significant (p=.08). | 56%,  10/18. |
| Jander et al. (2016). | N= 824.  Mean age: 16.  Country: Netherlands.  Inclusion criteria: Dutch school students, aged 15 to 19.  Exclusion criteria: None. | Name: Alcohol Alert.  Device: Computer.  Duration: Three sessions of the game ‘What Happened?’. No time limit.  Aim: To reduce binge drinking and overall consumption in Dutch adolescents.  Control: Waiting list control. | N= 9.  GSB, AP, RBG, C, FOB, SB, SOB, ISEC, SC. | Open-ended questions on binge drinking and weekly consumption. | Main author is the director of a company that licenses online health tools. | No significant effects were found on reported weekly consumption or binge drinking. | 63%,  19/30. |
| Jo et al. (2011). | N= 1,399.  Mean age: 32.  Country: Korea.  Inclusion criteria: Aged 20 to 40, South Korean residents, scored over 3/4 on AUDIT-C.  Exclusion criteria: Currently enrolled, or previously enrolled, in other alcohol treatment. | Name: On-BEAM (Online-based Brief Empowerment Program for Alcohol-use Monitoring).  Device: Computer.  Duration:  Aim: To reduce drinking behaviour in high-risk drinkers.  Control: Assessment only. | N= 8.  GSB, FOB, SB, SOB, IHC, ISEC, IEC, SC. | TLFB, AUDIT-C. | Had statistical power at baseline, however the sample size no longer met requirements at follow-up due to high attrition. | Intervention group reported significantly lower alcohol consumption than control (p=.012), significantly less binge drinking (p<.001), and lower AUDIT-C scores (p=.009). | 86%,  26/30. |
| Kypri et al. (2008). | N= 360.  Mean age: 20.  Country: New Zealand.  Inclusion criteria: Healthcare university student, studying in Australia, aged 17 to 20.  Exclusion criteria: None. | Name: eSBI (Electronic Screening and Brief Intervention).  Device: Computer.  Duration: 9 minutes for assessment. Time with feedback was not controlled.  Aim: To reduce hazardous drinking and assess the role of feedback.  Control: Booklet about the health effects of alcohol or waiting list control. | N= 7.  FOB, SB, SOB, B, FOO, SC, F/R. | AUDIT, TLFB, Alcohol Problems Scale. |  | Intervention led to greater reduction than controls in drinking frequency (p=.008) and overall drinking consumption (p=.020). Overall intervention effect remained significant at 12 month follow up (p=.010). | 67%,  20/30. |
| Kypri et al. (2009). | N= 1,578.  Mean age: 20.  Country: Australia.  Inclusion criteria: Aged 17 to 24, scored over 8 on AUDIT, enrolled in a university.  Exclusion criteria: None. | Name: THRIVE (Tertiary Health Research Intervention via Email Device).  Device: Computers:  Duration:  Aim: To reduce overall drinking behaviour in an at-risk population.  Control: Assessment only. | N= 7.  SB, SOB, B, IPB, IHC, ISEC, SC. | AUDIT, TLFB, second-hand effects of alcohol (Langley et al.), opinions on beverage labelling (Kypri et al.). |  | Intervention led to significantly lower drinking frequencies (p<.001), drinks consumed per occasion (p=.02) and total consumption (p<.001) compared the to control group. At 6 months, these effects were non-significant. | 60%,  18/30. |
| Kypri et al. (2012). | N= 1,415.  Mean age: 20.  Country: New Zealand.  Inclusion criteria: Aged 17 to 24, enrolled in one of eight universities, reported harmful of hazardous drinking on AUDIT.  Exclusion criteria: None. | Name: eSBI.  Device: Computer.  Duration: Mean of 10 minutes to complete and read feedback, although time was not limited.  Aim: To reduce hazardous alcohol consumption in students.  Control: Assessment only. | N= 9.  FOB, SB, SOB, B, FOO, IPB, IHC, ISEC, SC. | TLFB, AUDIT-C, Leeds Dependency Questionnaire (LDQ). |  | Intervention led to a significantly greater reduction in frequency of drinking (p=.01), typical quantity per occasion (p=.04), overall alcohol consumption (p<.001) and academic problems (p=.01). | 67%,  20/30. |
| Palfai et al. (2014). | N= 705.  Mean age: 18.  Country: US.  Inclusion criteria:  First year university students, over 18 years old.  Exclusion criteria: None. | Name: Feedback alcohol intervention.  Device: Computer.  Duration: 15-minute assessment. Time reading feedback was not controlled.  Aim: To prevent hazardous alcohol use in first year students.  Control: Received feedback about other health-related behaviours including sleep and nutrition. | N= 9.  FOB, B, IPB, IHC, ISEC, IEC, SC, BS, IB. | AUDIT, TLFB, YA-ACQ. |  | The intervention had no significant effect on binge drinking frequency. There was no significant effect on frequency on drinking-related consequences or total alcohol consequences. | 83%,  25/30. |
| Paschall et al. (2011). | N= 5,074.  Mean age: 19.  Country: US.  Inclusion criteria: First year university students, aged 18 to 20, from the US.  Exclusion criteria: None. | Name: AlcoholEdu.  Device: Computer.  Duration: Two to three hours.  Aim: To reduce alcohol misuse in students.  Control: Waiting list control. | N= 8.  GSB, PS, AP, SB, B, IHC, ISEC, SC. | TLFB. |  | No significant overall intervention effect. Participation in the intervention was significantly associated with greater binge drinking frequency reduction (p<.05) and alcohol use frequency reduction (p<.05). | 73%,  22/30. |
| Pederson et al. (2017). | N= 784  Mean age: 29.  Country: US.  Inclusion criteria: Veterans aged 18 to 34, scored above 3/4 on AUDIT.  Exclusion criteria: None. | Name: PNF (personalised normative feedback) condition.  Device: Any with internet access.  Duration: Amount of time with feedback was not regulated.  Aim: To reduce general alcohol use, motivation and perceived likelihood of consumption.  Control: Assessed on video game playing behaviour and received PNF. | N= 3.  FOB, SC, IB. | DDQ, Brief Young Adult Alcohol Related Questionnaire (YA-ARQ), Drinking Norms Rating Form (DNRF). | Participants received $45 after study completion. | Intervention led to a significant decrease in all variables at one month follow up (p<.05). The effect of the intervention was mediated by pre-intervention drinking severity (p=.04), with more severe drinkers reporting a greater reduction in consumption. | 70%,  20/30. |
| Possemato et al. (2019). | N= 30  Mean age: 30.  Country: US.  Inclusion criteria: Iraq or Afghanistan veterans, diagnosable or subthreshold PTSD, AUDIT score >7/8 (female/male).  Exclusion criteria: Enrolled in other treatment in the last 2 months, intentions to harm themselves. | Name: Thinking Forward.  Device: Computer.  Duration: 12 weeks, 8 hours of content in total.  Aim: To reduce both PSTD symptomology and hazardous alcohol use.  Control: Peer-supported versus self-managed Thinking Forward. | N= 11.  PS, AP, SB, SOB, SSU, IPB, IAA, ISEC, BPR, PAC, RNE. | Clinical Administered PTSD Scale (CAPS),  Alcohol Use Disorders Identification Test (AUDIT),  Timeline Follow-Back Interview (TLFB). | Paper affiliated with the intervention developers. | No significant differences found between groups. Higher participation in intervention was significantly associated with a greater decrease in drinking days per month (p=.026). | 73%,  22/30. |
| Schulz et al. (2013). | N= 448  Mean age: 42.  Country: Germany.  Inclusion criteria: Access to internet, aged 18 and over, and had an unhealthy drinking pattern (>7 on AUDIT)  Exclusion criteria: self or partner pregnant or trying to conceive. | Name: Alkohol-Alles im grünen Bereich?! (Translates to Alcohol- Everything within the limits?!)  Device: Computer  Duration: 3 sessions  Aim: Reduce alcohol intake in high-risk adult drinkers.  Control: Assessment only. | N= 7.  PS, AP, FOB, IHC, ISEC, SC, PAC. | Dutch 5-item Quantity-Frequency-Variability (QFV), AUDIT, Self-Report Habit Index (SRHI). |  | The intervention group showed a mean reduction of 3.9 drinks per week to .4 drinks per week, however this was not significant (p=.05). This effect remained non-significant when ITT analysis was performed. | 57%, 17/30. |
| Sinadinovic et al. (2014). | N= 476.  Mean age: 44.  Country: Sweden.  Inclusion criteria: Aged 15 and over, report at least hazardous alcohol use (>6/8 on AUDIT), reported on illicit drug use.  Exclusion criteria: None. | Name: Alkoholhjalpen.  Device: Computer.  Duration: Open-ended access to feedback.  Aim: To reduce problematic alcohol use and compare the efficacy of intervention types.  Control: eScreen brief normative feedback intervention. | N= 6.  PS, SB, IPB, IAA, ISEC, BS. | AUDIT, Drug Use Disorders Identification Test (DUDIT), health consequences questionnaire (not validated). |  | A significant decrease was found in AUDIT scores in both interventions (p<.001). The Alkoholhjalpen group reported significantly lower AUDIT scores than eScreen (p=.048). | 70%,  21/30. |
| Spijlkerman et al. (2010). | N= 278.  Mean age: 18.  Country: Netherlands.  Inclusion criteria: Ages 15 to 20, University of Maastricht students, reported frequent episodes of binge drinking.  Exclusion criteria: None. | Name: Intervention with normative feedback.  Device: Computer.  Duration: 15 minutes for intervention, time reading feedback was not controlled.  Aim: To reduce alcohol use in binge drinkers.  Control: Assessment only. | N= 7.  FOB, SB, SOB, IPB, IHC, SC, IB. | Alcohol Weekly Recall (AWR- Dutch version), TLFB. |  | Intervention significantly decreased weekly drinking in both groups at one month (p=.008; p=.05), and in the normative feedback group at 3 months follow up (p=.04).  Gender mediated normative feedback effects. | 80%,  24/30. |
| Suffoletto et al. (2014). | N= 765.  Mean age: 22.  Country: US.  Inclusion criteria: Aged 18 to 25, medically stable, had visited the emergency department in the last year, scored over 3/4 on AUDIT.  Exclusion criteria: Enrolled in other treatment, enrolled in high school. | Name: SMS assessment and feedback (SA+F) condition.  Device: Mobile phone.  Duration: 12 weeks.  Aim: To reduce alcohol consumption- specifically binge drinking behaviour.  Control: Assessment only. | N= 11.  GSB, PS, RBG, C, SB, IPB, IHC, SC, PC, BS, AEB. | Alcohol Smoking and Substance Involvement Screening Test (ASSIST), TLFB. | Participants paid $20 for participation. | Intervention led to a significant decrease in binge drinking days and drinks consumed on a drinking day. Those in the control group (with no feedback) showed an increase in drinking behaviour. | 83%,  25/30. |
| Voogt et al. (2013a). | N= 907.  Mean age: 21.  Country: Netherlands.  Inclusion criteria: Aged 18 to 24, reported heavy drinking (>21 drinks per week or >5 on one occasion), motivated to change behaviour.  Exclusion criteria: No internet access. | Name: What Do You Drink?  Device: Computer.  Duration: 20 minutes.  Aim: To reduce the frequency of overall drinking, binge drinking occasions and heavy drinking status.  Control: Assessment only. | N= 10.  GSB, PS, AP, FOB, SB, IPB, IAA, IHC, ISEC, SC. | Ecological momentary assessment (EMA) using TLFB. |  | Overall intervention effects were non-significant. Although, binge drinking was significantly reduced on weeks 1, 2, 7 and 12, and heavy drinking status on weeks 1, 2, 7 and 16. | 73%,  22/30. |
| Voogt et al. (2013b). | N= 828.  Mean age: 21.  Country: Netherlands.  Inclusion criteria: Aged 18 to 24, reported heavy drinking (>21 drinks per week or >5 on one occasion), reported motivation to change behaviour.  Exclusion criteria: No internet access. | Name: What Do You Drink?  Device: Computer.  Duration: 20 minutes.  Aim: To reduce heavy drinking in students and identify factors impacting drinking.  Control: Assessment only. | N= 10.  GSB, PS, AP, FOB, SB, IPB, IAA, IHC, ISEC, SC. | EMA using TLFB. | Research funded by the Netherlands Organisation for Health and Research Development. | At 1 month and 6 month follow ups there were no significant differences between intervention and control groups in heavy drinking, frequency of heavy drinking or weekly alcohol consumption. | 77%,  23/30. |
| Wallace et al. (2011). | N= 854.  Mean age: 38.  Country: UK.  Inclusion criteria: New users to Down Your Drink website, scored over 5 on AUDIT, over 18 years old.  Exclusion criteria: No internet access. | Name: Down Your Drink.  Device: Computer.  Duration: 45 minutes.  Aim: To reduce drinking and drinking-related problems.  Control: Directed to a different website which highlighted alcohol consequences but had no interactive features. | N= 9.  GSB, PS, SB, SOB, IPB, IAA, ISEC, SC, PAC. | TOT-AL, European Quality of Life tool (EQ-5D). | Research funded by the National Preventative Research Initiative and Alcohol Education Research Council. | The intervention showed no significant reductions in alcohol consumption or consequences. There were no significant differences between intervention or control groups. | 83%,  25/30. |
| Wilks et al. (2018). | N= 59  Mean age: 38.  Country: US.  Inclusion criteria: At least two heavy episodic drinking (HED) episodes in the last month, suicidal ideations present (>1 item on SBQ-R), show high emotional dysregulation (>46 on DERS).  Exclusion criteria: Enrolled in other treatment. | Name: Internet-delivered dialectical behaviour therapy skills training (iDBT-ST) condition.  Device: Computer.  Duration: 8 weeks, 30 to 50 minutes per week.  Aim: To reduce heavy episodic drinking and suicidal ideation.  Control: Waiting list control. | N= 12.  GSB, PS, AP, SB, SOB, IPB, IAA, MEC, RAS, BS, PAC, RNE. | TLFB, DERS, AUDIT, Scale for Suicidal Ideation (SSI). | Participants received $120 for study participation. Research funded by the National Institute of Alcohol Abuse and Alcoholism (NIAAA). | Intervention group drank significantly less alcoholic drinks than control group from baseline to two months post-intervention (p=.02). No significant differences were found in the intervention groups’ SSI, DERS or AUDIT scores. | 73%,  22/30. |
