# Supplementary material for: Identification of Behavior Change Techniques From Successful Web-Based Interventions Targeting Alcohol Consumption, Binge Eating, and Gambling: Systematic Review
Source: J Med Internet Res. 2021 Feb 9;23(2):e22694. doi: 10.2196/22694 (PMC7902193; doi:10.2196/22694)
Supplement: Multimedia Appendix 3 [file jmir_v23i2e22694_app3.docx]

Multimedia Appendix 3. Study characteristics for eligible studies which targeted Gambling.

| Authors | Participants | Intervention and control | BCTs | Outcome measures | Notes | Results | Risk of bias |
| --- | --- | --- | --- | --- | --- | --- | --- |
| Bücker et al. (2018). | N= 140.  Mean age: 36.  Country: Germany.  Inclusion criteria: Self-reported problem gambler, aged 18 to 65.  Exclusion criteria: Reported feelings of suicide, diagnosis of bipolar disorder or psychosis. | Name: Deprexis.  Device: Computer or mobile phone.  Duration: 8 weeks, 1 hour per week.  Aim: To reduce depression scores and slot machine behaviour.  Control: Waiting list control. | N= 8.  GSB, PS, C, SB, IPB, IAA, R, RNE. | Web Screening Questionnaire (WSQ), Patient Health Questionnaire (PHQ-9), South Oaks Gambling Screen (SOGS), Pathological Gambling Adaption of Yale-Brown Obsessive-Compulsive Scale (PG-YBOCS). | Had statistical power at baseline, however the sample size no longer met requirements at follow-up due to high attrition. Research funded by a German gaming and gambling company. | Per protocol analysis showed no significant intervention effects. ITT analysis showed a significant decrease in depressive symptoms (p<.001), gambling behaviour (p=.005) and pathological gambling (p=.023). When MI was used to impute data, depressive symptoms remained significant (p=.03), but gambling behaviour and pathological gambling were no longer significant. | 73%,  22/30. |
| Canale et al. (2016). | N= 168.  Mean age: 15.  Country: Italy.  Inclusion criteria: Enrolled in a pre-selected Italian high school.  Exclusion criteria: None. | Name: Web-based intervention (WBI) group.  Device: Computer.  Duration: 4 weeks.  Aim: To reduce gambling rates in students and educate them on gambling related consequences.  Control: Assessment and personalised normative feedback provided. | N= 7.  FOB, SB, IPB, IHC, ISEC, IEC, SC. | SOGS, GAS, TLFB. |  | Intervention showed no significant intervention effects on gambling frequency, attitudes and expenditure. Intervention group showed a significant decrease in gambling severity scores (p<.05), although there were no differences found when compared to the control group. | 63%,  19/30. |
| Casey et al. (2017). | N= 174.  Mean age: 44.  Country: Australia.  Inclusion criteria: Aged 18 and over, met DSM criteria for pathological gambling.  Exclusion criteria: Enrolled in other gambling treatment, at risk of suicide or psychotic disorder. | Name: I-CBT (Internet-Based Cognitive Behavioural Therapy).  Device: Computer.  Duration: 6 weeks.  Aim: To reduce problem gambling.  Control: Waiting list control and active control of monitoring, feedback and support intervention. | N= 7.  PS, FOB, IAA, E, BS, RNE, IB. | SOGS, Gambling Symptom Assessment Scale (GSAS), Gambling Urge Scale (GUS), Gambling Refusal Self-Efficacy Questionnaire (GRSEQ), Gambling Related Cognitions Scale (GRCS), DASS, AUDIT. |  | Compared to the control, the intervention showed significantly greater reduction in overall gambling severity (p<.001), gambling frequency (p<.001), gambling urge (p<.001) and gambling related consequences (p<.001). Comparing intervention groups, CBT showed significantly greater reductions in urges (p<.001) and stress (p<.001). | 80%,  24/30. |
| Hodgins et al. (2019). | N= 123.  Mean age: 47.  Country: Canada.  Inclusion criteria: Over the age of 18, reported gambling problem (<3 on PGSI), had gambled in last 30 days.  Exclusion criteria: Enrolled in other gambling treatment. | Name: Check Your Gambling.  Device: Computer.  Duration: Time with feedback was not controlled.  Aim: To reduce gambling and related behaviour in problem gamblers.  Control: Received self-management tools. | N= 3.  FOB, SB, IPB. | Problem Gambling Severity Index (PGSI), NORC DSM Screen for Gambling Problems (NODS), TLFB. |  | Intervention successfully reduced gambling behaviour which was maintained at 12 months (p<.05). However, there were no significant differences between intervention group and control group in any measures. | 90%,  27/30. |
| Ivanova, Magnusson and Carlbring (2019). | N= 4,328.  Mean age: 29.  Country: Finland.  Inclusion criteria: Preregistered on gambling platform, over 18 years old, online slot games were most frequently used gambling method for 80% of days.  Exclusion criteria: None. | Name: Drop-limit prompt group.  Device: Computer.  Duration: 90 days if the prompt was not removed.  Aim: To reduce the amount spent by online gamblers by using deposit limit prompts.  Control: Behaviour monitored with no deposit prompts. | N= 2.  GSB, RPE. | Online behaviour was monitored with total sum of bets placed and winnings measured, as well as one’s tendency to set, remove or increase a deposit limit. |  | Intervention groups set more limits compared to controls. Men were more likely than women to do so (p=.005). No significant effects were found on net loss of gamblers. When only the top 10% of gamblers were assessed, this remained non-significant. | 67%,  20/30. |
| Neighbors et al. (2015). | N= 226.  Mean age: 23.  Country: US.  Inclusion criteria: Over the age of 18, enrolled on one of two pre-selected universities, reported gambling issues (>2 on SOGS).  Exclusion criteria: None. | Name: Computer-delivered personalised normative feedback.  Device: Computer.  Duration: One hour to complete. Feedback viewed for roughly 5 minutes, although this was not controlled.  Aim: To reduce gambling behaviour in student problem-gamblers.  Control: Received attention control feedback. | N= 6.  FOB, SB, SOB, ISEC, SC, IB. | SOGS, Gambling Quantity and Perceived Norms Scale (GQPNS), Gambling Problems Index (GPI), Identification with groups measures (adaptation of Rocca et al.). |  | Intervention had a significant effect on money lost to gambling (p=.005) and gambling problems (p=.016). At 6 month follow up money lost to gambling remained significant (p<.001), but gambling problem scores did not. Changes in perceived norms mediated gambling losses at 6 months. | 83%,  25/30. |
| Wood and Wohl (2015). | N= 1,558.  Mean age: Missing.  Country: Sweden.  Inclusion criteria: Enrolled in Svenska Spel betting website.  Exclusion criteria: None. | Name: Playscan.  Device: Computer.  Duration: Time with feedback was not controlled. Online behaviour was assessed for 10 weeks.  Aim: To reduce gambling and spending in participants and investigate if effects differed with gambling severity.  Control: Behaviour monitored with no feedback. | N= 2.  FOB, FOO. | Online behaviour was monitored with amount deposited, won and lost measured. |  | There were no significant intervention effects on behaviour. However, the intervention did show significantly less money deposited in low-risk only players compared to the low-risk control group (p<.001). | 61%,  11/18. |
