# Supplementary material for: Identification of Behavior Change Techniques From Successful Web-Based Interventions Targeting Alcohol Consumption, Binge Eating, and Gambling: Systematic Review
Source: J Med Internet Res. 2021 Feb 9;23(2):e22694. doi: 10.2196/22694 (PMC7902193; doi:10.2196/22694)
Supplement: Multimedia Appendix 4 [file jmir_v23i2e22694_app4.docx]

Multimedia Appendix 4. Study characteristics for eligible studies which targeted Binge eating.

| Authors | Participants | Intervention and control | BCTs | Outcome measures | Notes | Results | Risk of bias |
| --- | --- | --- | --- | --- | --- | --- | --- |
| Boucher et al. (2016). | N= 40.  Mean age: 45.  Country: New Zealand.  Inclusion criteria: Female, aged 40 to 50, New Zealand resident, overweight (BMI), below average score on Intuitive Eating Scale (<65), able to engage in light exercise.  Exclusion criteria: None. | Name: Mind, Body, Food.  Device: Computer.  Duration: 14 weeks consisting of 12 modules.  Aim: To increase intuitive eating in overweight women.  Control: No control group. | N= 10.  PS, SB, SOB, IPB, SC, BPR, BS, CIFO, RNE, F/R. | BMI. Intuitive Eating Scale (IES-S), two items of EDE-Q. |  | Intervention significantly increased intuitive eating scores (p<.001; p<.01 for all four subscales). | 61%,  11/18. |
| Carrard et al. (2011). | N= 58.  Mean age: 36.  Country: France.  Inclusion criteria: Female, aged 18 to 60, reported at least one binge episode per week in the last three months.  Exclusion criteria: Previous attempt of suicide, had undergone weight management surgery. | Name: Internet Self-Help programme.  Device: Computer.  Duration: 11 modules over 6 months.  Aim: To reduce binge eating and bulimic behaviour in participants.  Control: Waiting list control. | N= 9.  PS, FOB, SB, IAA, MEC, E, BPR, PAC, CIFO. | Eating Disorders in Obesity Questionnaire (EDO), EDE-Q, EDI, Three-Factor Eating Questionnaire (TFEQ). |  | Using ITT to impute missing data, the intervention led to a significantly greater decrease the bulimia subscale than the control group (p<.001). Drive for thinness, body dissatisfaction, shape concerns, number of binge episodes and hunger scores all significantly improved from baseline to post intervention (p<.05). All other measures were non-significant. | 70%,  21/30. |
| Lyzwinski et al. (2019). | N= 72.  Mean age: 20.  Country: New Zealand.  Inclusion criteria: Queensland university student, aged 18 to 25, BMI above healthy threshold (>20).  Exclusion criteria: Pregnant, history of psychiatric illness. | Name: My Student Mindfulness App.  Device: Mobile phone.  Duration: 11 weeks.  Aim: To reduce weight, weight-related behaviours and stress.  Control: Completed a behavioural self-monitoring diary for diet and exercise. | N= 8.  PS, IPB, IAA, DOB, BS, HR, GT, RNE. | Physical activity (IPAQ), TFEQ, Perceived Stress Scale (PSS), Mindfulness Eating Questionnaire (MEQ). |  | Intervention group had significantly higher mindfulness eating scores (p<.01) and binge eating scores (p<.05). | 70%,  21/30. |
| Ruwaard et al. (2013). | N= 105.  Mean age: 31.  Country: Netherlands.  Inclusion criteria: Aged 16 and over, females, self-reports of binge eating, inappropriate weight management or concern over body shape.  Exclusion criteria: Suicidal ideations present, indication of a psychological disorder. | Name: Online Cognitive-Behavioural treatment.  Device: Computer.  Duration: 20 weeks with 25 items of feedback.  Aim: To prevent bulimic symptoms from progressing in individuals at risk.  Control: Waiting list condition. | N= 8.  AP, SB, IAA, BE, BS, O, AEB, VSI. | EDE-Q, Body Attitudes Test (BAT), Depression Anxiety Stress Scale (DASS), Dissociation Questionnaire, Self-Harm Inventory. | Had statistical power at baseline, however the sample size no longer met requirements at follow-up due to high attrition. | Intervention led to a significantly greater decrease in bulimic symptoms (p=.04) compared to controls. | 67%,  20/30. |
| Svensson et al. (2014). | N= 620.  Mean age: 40.  Country: Sweden.  Inclusion criteria: Pre-registered to Swedish weight loss program.  Exclusion criteria: Reported unrealistic BMI, reported unrealistic weight loss goal. | Name: Viktklubb.  Device: Computer.  Duration: 3, 6, or 12 month plans.  Aim: To reduce emotional, uncontrolled and cognitively restrained eating. To assess the impact of gender on eating behaviour and intervention success.  Control: No control group. | N= 9.  FOB, SB, SOB, FOO, SSU, IPB, SC, BS, CS. | TFEQ. |  | No significant time x sex interactions for uncontrolled eating behaviour or cognitively restrained eating. Time variable was significant with participants showing decreased uncontrolled eating (p<.001) cognitively restrained eating (p<.001). A significant time x sex interaction was found for binge eating (p<.001), with the effect of time significant in men (p<.001) but not women. | 78%,  14/18. |
| Völker, Jacobi and Barr Taylor (2011). | N= 22.  Mean age: 26.  Country: Germany.  Inclusion criteria: aged 18 to 38, showed weight or shape concerns (>47 on WCS), showed at least one symptom of disordered eating (SLC-90)  Exclusion criteria: Signs of severe depression (BDI). | Name: Student Bodies (adapted version).  Device: Computer.  Duration: 8 weeks.  Aim: To prevent eating disorders in women with subclinical symptoms.  Control: No control group. | N= 8.  PS, FOB, SB, SOB, ISEC, MEC, BS, VSI. | Weight and Shape Concerns (WSC), Structured Clinical Interview for DSM (SCID Module H- Eating Disorders), Eating Disorders Inventory (EDI), Eating Disorders Examination Questionnaire (EDE-Q), TLFB. |  | Intervention found no significant effect on overall scores, or bulimia scores. Although the weekly TLFB measures showed a significant decrease in binges and restrictive eating (p=.031). | 50%,  9/18. |

*References within the table: Andrade et al. (2016) [56]; Arnaud et al. (2016) [57]; Bertholet et al. (2015) [58]; Bertholet et al. (2017) [59]; Bewick et al. (2010) [60]; Bewick et al. (2013) [61]; Bingham et al. (2011) [62] Crombie et al. (2018) [53]; Deady et al. (2016) [63]; Doumas and Hausviet (2008) [64]; Doumas et al. (2011) [65]; Dulin, Gonzalez and Campbell (2014) [66]; Fazzino, Rose and Helzer (2016) [67]; Ganz et al. (2018) [68]; Haug et al. (2013) [69]; Jander et al. (2016) [70]; Jo et al. (2011) [71]; Kypri et al. (2008) [72]; Kypri et al. (2009) [73]; Kypri et al. (2012) [74]; Palfai et al. (2014) [75]; Paschall et al (2011) [76]; Pederson et al. (2017) [54]; Possemato et al. (2019) [77]; Schulz et al. (2013)[78]; Sinadinovic et al. (2014) [79]; Spijlkerman et al. (2010) [80]; Suffoletto et al. (2014) [81]; Voogt et al. (2013a) [82]; Voogt et al. (2013b) [83]; Wallace et al. (2011) [84]; Wilks et al. (2018) [85]; Bücker et al. (2018) [86]; Canale et al. (2016) [87]; Casey et al. (2017) [88]; Hodgins et al. (2019) [89]; Ivanova, Magnusson and Carlbring (2019) [90]; Neighbors et al. (2015) [91]; Wood and Wohl (2015) [92]; Boucher et al. (2016) [93]; Carrard et al. (2011) [42]; Lyzwinski et al. (2019) [95]; Ruwaard et al. (2013) [96]; Svensson et al. (2014) [97]; Völker, Jacobi and Barr Taylor (2011) [98].*

*BCT Key (Item from BCT Taxonomy v1 [45], Acronym from the BCT Periodic Table [99]; 1.1 Goal setting (behaviour), GSB; 1.2 Problem solving, PS; 1.4 Action Planning, AP;1.5 Review behaviour goal(s), RBG; 1.9 Commitment, C; 2.2 Feedback on behaviour, FOB; 2.3 Self-monitoring of behaviour, SB; 2.4 Self-monitoring of outcome(s) of behaviour, SOB; 2.6 Biofeedback, B; 2.7 Feedback on outcome(s) of behaviour, FOO; 3.1 Social support (unspecified), SSU; 4.1 Instruction on how to perform the behaviour, IPB; 4.2 Information about antecedents, IAA; 4.3 Re-attribution, R; 4.4 Behavioural experiments, BE; 5.1 Information about health consequences, IHC; 5.3 Information about social and environmental consequences, ISEC; 5.4 Monitoring of emotional consequences, MEC; 5.6 Information about emotional consequences, IEC; 6.1 Demonstration of the behaviour, DOB; 6.2 Social Comparison, SC; 7.1 Prompts or cues, PC; 7.5 Remove aversive stimulus, RAS; 7.7 Exposure, E; 8.1 Behavioural practice/rehearsal, BPR; 8.2 Behaviour substitution, BS; 8.4 Habit reversal, HR; 8.5 Overcorrection, O; 8.7 Graded tasks, GT; 9.1 Credible source, CS; 9.2 Pros and cons, PAC; 9.3 Comparative imagining of future outcomes, CIFO; 10.2 Material reward (behaviour), MRB; 11.2 Reduce negative emotions, RNE; 12.1 Restructuring the physical environment, RPE; 12.3 Avoidance/reducing exposure to cues for the behaviour, AEB; 13.2 Framing/reframing, F/R; 13.3 Incompatible beliefs, IB; 13.4 Values self-identity, VSI.*
